# Causal Effect of Macronutrient and Micronutrient Intake on Stroke: A Two-Sample Mendelian Randomization Study

**DOI:** 10.3390/nu16172818

**Published:** 2024-08-23

**Authors:** Guozhang Dong, Wanqian Xu, Lin Xu

**Affiliations:** 1 The Fourth School of Clinical Medicine, Nanjing Medical University, Nanjing 210009, China; dgz_1995@foxmail.com; 2 Department of Public Health and Medical Technology, Xiamen Medical College, Xiamen 361023, China; wanqianxu@outlook.com

**Keywords:** macronutrients, micronutrients, stroke, mendelian randomization, causality

## Abstract

(1) Background: Estimating the causal association between nutrient intake, as a modifiable risk factor, and stroke risk is beneficial for the prevention and management of stroke. However, observational studies are unavoidably influenced by confounding factors and reverse causation. (2) Methods: We performed a two-sample Mendelian randomization (MR) to estimate the effects of nutrient intake on stroke risk. Summary statistics for nutrients, including 4 macronutrients and 14 micronutrients, were derived from 15 genome-wide association studies (GWAS). Data on stroke and its subtypes were sourced from the MEGASTROKE consortium. (3) Results: Genetically predicted magnesium levels, as the protective factors, were significantly associated with a lower risk of cardioembolic stroke (OR: 0.011, 95% CI: 0–0.25, *p*-value: 0.005) in the IVW method. Additionally, vitamin C reduced the risk of cardioembolic stroke (OR: 0.759, 95% CI: 0.609–0.946, *p*-value: 0.014) and vitamin B9 reduced the risk of small vessel stroke (OR: 0.574, 95% CI: 0.393–0.839, *p*-value: 0.004) with the IVW method. However, the association of vitamin B6 with an increased risk of large-artery stroke (OR: 1.546, 95% CI: 1.009–2.37, *p*-value: 0.046) in the Wald ratio method should be interpreted cautiously due to the limited number of SNPs. There was also suggestive evidence that magnesium might decrease the risk of both any stroke and ischemic stroke. (4) Conclusions: Our MR analysis highlights the protective roles of magnesium, vitamin C, and vitamin B9 in stroke prevention, making them key targets for public health strategies. However, the findings related to vitamin B6 are less certain and require further validation.

## 1. Introduction

Stroke has emerged as a significant global health issue, leading to widespread disability and death. It is primarily caused by brain infarction (ischemic stroke) and, less frequently, by intracerebral hemorrhage (ICH). Ischemic stroke has three common etiological subtypes: large-artery stroke, small-vessel stroke, and cardioembolic stroke [1]. With the increasing global burden of stroke, the prevention and management of stroke are critical public health priorities [2].

Modifiable factors like diet and lifestyle play a vital role in stroke prevention or development, which is widely accepted by the public [3,4]. Many studies have shown that dietary factors have been implicated in stroke risk modulation [3,5]. A large meta-analysis of 122 prospective observational studies revealed the impact of dietary factors related to food groups, foods, macronutrients, and micronutrients on stroke risk. Specifically, increased intake of fruits and vegetables reduced stroke risk, while red and processed meats increased the risk [6]. Moreover, adherence to dietary patterns such as the Mediterranean diet and the Dietary Approaches to Stop Hypertension (DASH) diet has been associated with lower stroke risk [7].

However, whole foods contain a complex mixture of nutrients, making it difficult to determine which specific components contribute to health outcomes, especially in observational studies that are unavoidably influenced by confounding factors and reverse causation. To solve the limitations, Mendelian randomization (MR) offers a robust approach to assess the causal effects of exposures on outcomes by using single-nucleotide polymorphisms (SNPs) as instrumental variables (IVs). This method leverages the random allocation of genes at conception, which mimics the randomization process in randomized controlled trials, thereby minimizing confounding and reverse causation biases. It is important that research can be conducted more easily by obtaining exposure and outcome data from separate, non-overlapping, mostly population-based, publicly available GWAS summary statistics for a two-sample MR analysis [8,9,10].

Therefore, we aim to conduct a two-sample Mendelian Randomization (MR) analysis to comprehensively evaluate the causal relationships between nutrient intake and stroke risk. This study will include a total of 18 macro- and micronutrients and examine various stroke outcomes such as any stroke, ischemic stroke, and its subtypes, including large-artery stroke, small-artery stroke, and cardioembolic stroke, thereby providing valuable insights for dietary recommendations and public health strategies to prevent stroke.

## 2. Methods

### 2.1. Study Design

**Figure 1:**
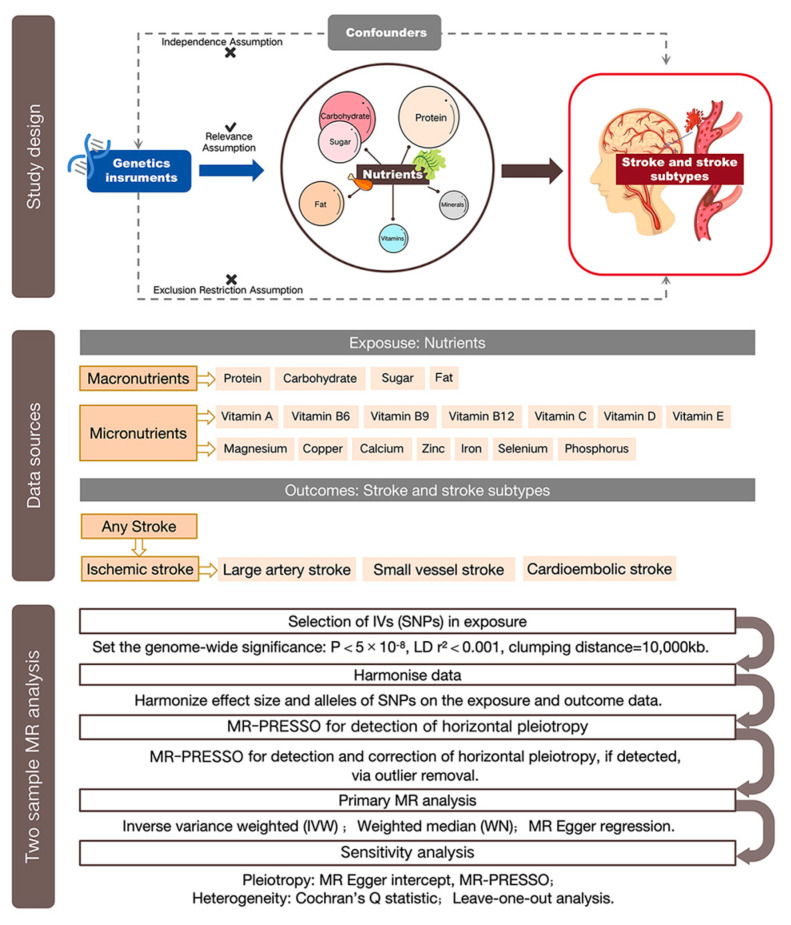
The study design, data sources, and flowchart of MR study on the causal association between nutrients and stroke. The study design section outlines the essential assumptions for SNPs to be valid instrumental variables (IVs): Relevance assumption; independence assumption; and exclusion restriction. The data sources section details the types of nutrients examined, encompassing both macro- and micronutrients, and the various stroke outcomes. The MR analysis flowchart section describes the process from SNP selection and data harmonization to the application of primary MR analytical methods, rounded off with sensitivity analyses to ensure the validity and robustness of the causal relationships identified. Abbreviations: LD, linkage disequilibrium, used to measure the correlations between SNPs; SNP, single-nucleotide polymorphism, as instrumental variables for the exposures and outcomes; MR, Mendelian randomization.

Based on two sets of GWAS summary statistics, a two-sample MR analysis was performed to assess the causal association between nutrient intake and stroke risk. SNPs associated with nutrients were selected as IVs and used to estimate the causal relationships. The included SNPs should fulfill the following assumptions to be valid IVs: (1) the IVs are linked to nutrients; (2) the IVs are unrelated to confounders; and (3) the IVs should only affect stroke through the nutrients. Our findings were released in accordance with the MR-STROBE guidance. The detailed flowchart of the study design and recruitment process is provided in Figure 1.

### 2.2. Data Source

#### 2.2.1. Macronutrients and Micronutrients

We searched the GWAS catalog, IEU Open GWAS project, and PubMed for the latest and largest genome-wide association studies (GWASs) conducted on dietary nutrients in European-descent participants, covering publications from inception to 1 January 2024. Macronutrient data (protein, carbohydrates, sugar, and fat) were obtained from a GWAS conducted by Meddens et al. [11] This study involved 268,922 participants aged 27 to 71 years who had completed 24 h self-reported dietary questionnaires that assessed the consumption of 70 or more food items. Macronutrient intake is expressed as a percentage of total energy intake (E%). In addition, a total of 14 micronutrients, including vitamins and minerals, were included: vitamin A, vitamin B6, vitamin B9, vitamin B12, vitamin C, vitamin D, vitamin E, selenium, copper, calcium, zinc, iron, magnesium, and phosphorus. Biochemical biomarkers in serum, plasma, and other specimens, which provide objective measures of nutrient status and bioavailability, are typically used to assess micronutrient intake (Table 1).

#### 2.2.2. Stroke

The GWAS database for stroke is from the Multi-ancestry Genome-wide Association Study of Stroke (MEGASTROKE) consortium, an international collaboration launched by the International Stroke Genetics Consortium (ISGC). This dataset is derived from a meta-analysis of 21 studies, covering about 8 million SNPs and indels in up to 67,162 stroke cases and 454,450 controls from various ancestries. In our study, we only included those of European descent, encompassing any stroke (n = 446,696), ischemic stroke (n = 440,328), and its subtypes such as large-artery stroke (n = 150,765), small-artery stroke (n = 198,048), and cardioembolic stroke (n = 211,763) (Table 1).

### 2.3. Selection Criteria of Instrumental Variables

Instrumental variables (IVs) were selected according to the following criteria: (1) SNPs should be associated with macro- and micronutrients at a genome-wide significance level (*p*-value < 5 × 10^−8^). (2) Independent SNPs were selected, with linkage disequilibrium r^2^ < 0.001 within a window of 10,000 kb. (3) The palindromic SNPs with intermediate allele frequencies were removed. (4) Weak instrument bias was assessed using F statistics. An F-statistic >10 was used to indicate sufficient strength of the selected SNPs. Detailed information on IVs for macro- and micronutrients is listed in Supplementary Table S1.

### 2.4. Statistical Analysis

We conducted a two-sample MR analysis to estimate the causal effect of macro- and micronutrient intake on stroke risk. The inverse variance weighted (IVW) approach was the main method to estimate causal effects. It is most effective when all genetic variants are valid instruments and there is no pleiotropy. Additionally, the weighted median (WM) method was employed to provide robust estimates even if up to 50% of the instruments were invalid. And the MR-Egger method was utilized to provide consistent causal estimates despite the presence of pleiotropy.

The MR-Egger regression intercept test and MR-PRESSO were employed as a sensitivity analysis to test horizontal pleiotropy. Among these, the MR-PRESSO is a method that systematically detects and corrects horizontal pleiotropic outliers in MR testing through three steps: the MR-PRESSO global test, the MR-PRESSO outlier test, and the MR-PRESSO distortion test. In addition, the robustness of the significant findings was further validated by detecting heterogeneity using Cochrane’s Q test and leave-one-out sensitivity analysis.

A significant causal relationship between nutrients and stroke was considered if the following criteria were satisfied: (1) The IVW method showed a significant result (*p*-value < 0.05); (2) the estimation directions of the IVW, weighted median, and MR-Egger methods were consistent; and (3) there was no evidence of pleiotropy or heterogeneity, as assessed by the MR-Egger regression intercept test, MR-PRESSO global tests, and Cochran’s Q test (all *p*-values > 0.05).

## 3. Results

**Figure 2:**
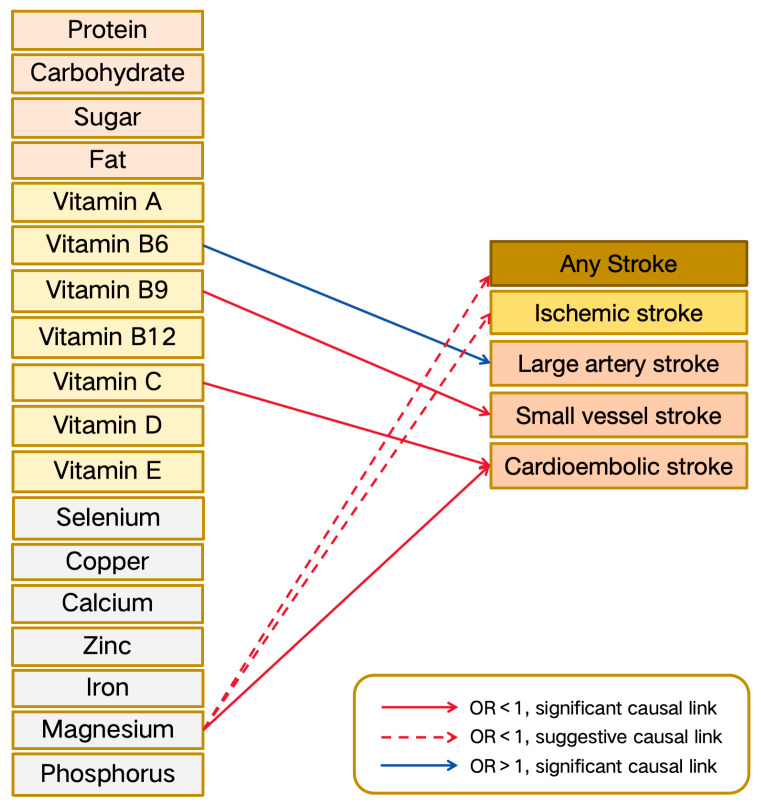
Overview of MR results for causal effects of nutrients on strokes. Different line styles and colors indicate the strength and direction of associations between nutrients and stroke outcomes: solid blue lines indicate significant increases in stroke risk, dashed blue lines indicate suggestive increases in stroke risk, solid red lines indicate significant decreases in stroke risk, and dashed red lines indicate suggestive decreases in stroke risk. Abbreviations: OR: odds ratio.

**Figure 3:**
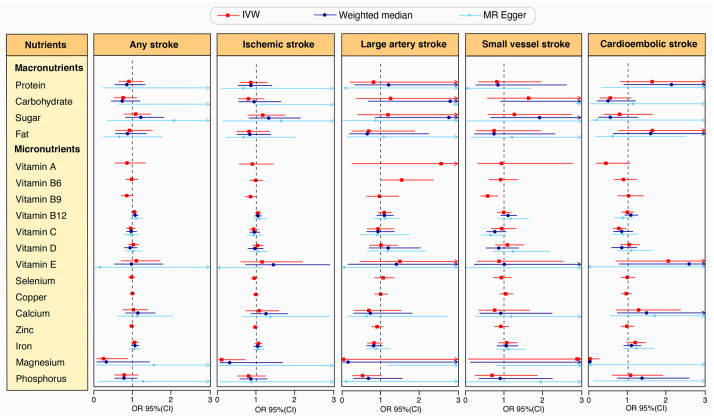
MR estimates the causal effects of nutrients on stroke using three analytical methods. Red squares represent IVW results, blue circles for weighted median, and light blue diamonds for MR-Egger. Points represent the estimated odds ratios (ORs), while horizontal lines indicate the 95% confidence intervals (CIs). An OR quantifies the association between a nutrient and stroke risk: OR >1 suggests increased risk, and OR <1 suggests reduced risk. Statistical significance is confirmed if the 95% CI does not include 1.

Our research findings revealed that magnesium levels were significantly associated with a reduced risk of cardioembolic stroke (OR: 0.011, 95% CI: 0–0.25, *p*-value: 0.005) using the IVW method, simultaneously supported by the weighted median method (OR: 0.002, 95% CI: 0–0.05, *p*-value: 0.0001) (Figure 2 and Figure 3, Supplementary Table S2).

Additionally, vitamin C levels were associated with a reduced risk of cardioembolic stroke (OR: 0.759, 95% CI: 0.609–0.946, *p*-value: 0.014) in the IVW method. Vitamin B9 levels also showed a protective effect, reducing the risk of small-vessel stroke (OR: 0.574, 95% CI: 0.393–0.839, *p*-value: 0.004) in the IVW method. Conversely, vitamin B6 levels were linked to an increased risk of large-artery stroke (OR: 1.546, 95% CI: 1.009–2.37, *p*-value: 0.046) in the Wald ratio method (Figure 2 and Figure 3, Supplementary Table S2).

There was suggestive evidence that magnesium levels reduced the risk of any stroke (OR: 0.249, 95% CI: 0.071–0.868, *p*-value: 0.029) and ischemic stroke (OR: 0.095, 95% CI: 0.013–0.713, *p*-value: 0.022) in the IVW method. However, the results should be considered as indicative and interpreted with caution due to inconsistencies in the MR-Egger estimates (Figure 2 and Figure 3, Supplementary Table S2).

**Figure 4:**
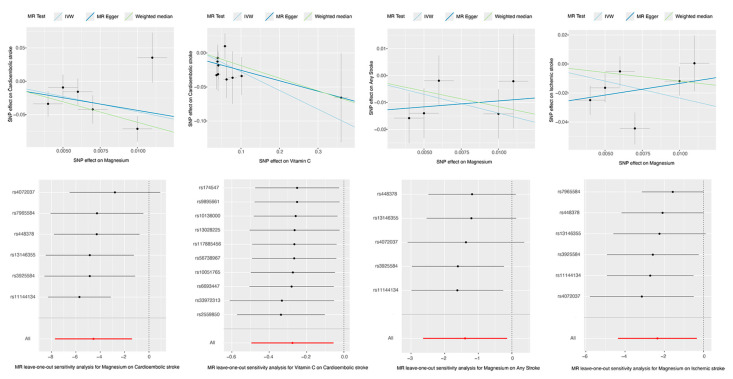
Scatter plot and leave-one-out analysis for the effects of nutrients on stroke. This figure illustrates scatter plots and leave-one-out sensitivity analyses for four of the seven identified causal relationships: the effects of magnesium on cardioembolic stroke, any stroke, and ischemic stroke, as well as the impact of vitamin C on cardioembolic stroke. The analyses for vitamins B6 and B9 are omitted due to a limited number of SNPs. The scatter plots show results from three MR methods: Inverse Variance Weighted (IVW), MR-Egger, and Weighted Median. The leave-one-out analysis re-estimates the overall effect by sequentially excluding one SNP at a time, with results displayed in a forest plot. In the forest plot, each black dot and line represent the IVW estimate and its 95% confidence intervals (CIs) after excluding one SNP, while the red dot and line indicate the IVW estimate using all SNPs. Abbreviations: SNP, single-nucleotide polymorphism; MR, Mendelian randomization; IVW, inverse variance weighted.

No evidence of pleiotropy among IVs was detected in our MR analysis, as the MR-Egger intercept and MR-PRESSO global tests were employed to remove outliers before analysis. Despite this, Cochran’s Q test indicated some heterogeneity among the IVs after adjustment, such as the suggestive causal relationship between magnesium levels and ischemic stroke risk, where heterogeneity was indicated (IVW test *p*-value: 0.023). Moreover, the leave-one-out analysis revealed that the result was not significantly affected by removing any single SNP. And the F-statistic ranged from 16.00 to 404.69. All results were above the criterion of >10, indicating that weak instrument bias was less likely to affect it (Figure 4, Supplementary Tables S1 and S2).

## 4. Discussion

Our findings, based on a two-sample MR analysis, provide robust evidence of causal relationships between specific nutrients and different types of strokes. Genetically predicted magnesium, vitamin C, and vitamin B9 were associated with a lower risk of various types of strokes. In contrast, vitamin B6 was linked to an increased risk of large-artery stroke. These results offer significant insights for both public health strategies and clinical interventions for stroke.

We found that magnesium was a significant protective factor against various types of strokes, particularly cardioembolic stroke, as is consistent with several previous studies. A meta-analysis of 122 prospective observational studies published on 24 May 2022 highlighted high- or moderate-certainty evidence supporting the inverse relationship between magnesium intake and ischemic stroke [6]. Another meta-analysis of prospective studies discovered that higher serum magnesium levels were strongly linked with a lower risk of cardiovascular disease, while higher dietary magnesium intake was associated with a reduced risk of ischemic heart disease [24]. Furthermore, a meta-analysis of seven prospective trials with 241,378 participants revealed that increasing daily magnesium intake by 100 mg reduced the overall risk of stroke by 8%, particularly ischemic stroke [25]. It has been shown that magnesium provides these protective effects by regulating blood pressure, reducing inflammation, and offering neuroprotection, which collectively lowers the risk of stroke [26,27]. Our findings contribute to the growing body of evidence supporting the cardiovascular benefits of magnesium and highlight the importance of magnesium intake through diet or supplementation to prevent stroke.

Numerous studies have indicated that Vitamin C, as a potent antioxidant, has shown effectiveness in reducing cardiovascular events, particularly strokes, consistent with our research findings. A meta-analysis of prospective cohort studies, including over 29,648 participants, found that individuals with higher circulating vitamin C levels had a significantly lower risk of stroke [28]. Another meta-analysis of 17 prospective cohort studies showed that higher levels of circulating vitamin C and higher dietary vitamin C intake were found to be inversely correlated with the incidence of stroke [29]. However, randomized controlled trials (RCTs) published in JAMA, involving over 14,000 older men, have not consistently shown significant long-term benefits of vitamin C supplementation alone for stroke prevention [30]. In addition, a meta-analysis of 10 trials also failed to show any evidence of an effect of vitamin C on the risk of stroke [31]. These discrepancies suggest that its efficacy is influenced by complex factors like study design, participant health status, adherence to supplementation, and the dosages used. This further emphasizes the benefit of our MR analysis to avoid confounding variables. Currently, the proposed mechanisms include Vitamin C’s antioxidant properties, reduction in oxidative stress, inhibition of low-density lipoprotein (LDL) oxidation, and enhancement of nitric oxide (NO) production, which collectively contribute to vascular health and reduce the likelihood of atherosclerosis and subsequent cardioembolic events [32,33]. Our research further confirmed that vitamin C supplementation was beneficial for preventing stroke, particularly cardioembolic stroke.

Vitamin B9 is one of the B vitamins, also known as folate or folic acid when used in supplements. A large-scale meta-analysis of 21 randomized clinical trials on folic acid supplementation and stroke prevention revealed that folic acid has a considerable overall advantage in terms of lowering stroke risk. The results highlight the significant impact of variables like grain fortification and a history of stroke or myocardial infarction on the effectiveness of folic acid for stroke prevention [34]. There were other studies indicating the positive effects of B vitamins on stroke prevention [35,36]. A comprehensive review revealed that supplementation with these B vitamins (vitamin B9, vitamin B12, and vitamin B6) can lower blood homocysteine concentrations by about 25% and reduce the relative risk of stroke by approximately 10% compared with a placebo [37]. Vitamin B supplementation significantly reduces stroke risk by lowering homocysteine levels, as several meta-analyses have confirmed [38,39]. Additionally, the VITATOPS (Vitamins to prevent stroke) trial revealed that, in patients with severe cerebral small-vessel disease (CSVD), vitamin B supplementation significantly reduced white matter hyperintensities (WMH), a marker of small-vessel disease progression [40]. Overall, there is substantial evidence supporting the beneficial effects of Vitamin B9 on stroke prevention. The possible mechanisms include the reduction in homocysteine levels, improvement of endothelial function, reduction in oxidative stress in blood vessels, etc. Most studies have focused on the general stroke population, but our research based on MR analysis specifically highlighted the impact of Vitamin B9 on small-vessel stroke, providing more new insights [37].

Multiple studies have demonstrated the benefits of B vitamins, including vitamin B6, for stroke prevention [35,36,37,41]. However, these studies included folic acid, vitamin B12, and other B vitamins to evaluate cardiovascular risk, making it challenging to attribute the observed benefits solely to vitamin B6. Conversely, our study found that vitamin B6 may increase the risk of large-artery stroke, which conflicts with these findings. Furthermore, some studies have demonstrated the ineffectiveness of vitamin B6 supplementation in the prevention of cardiovascular events, including stroke [42,43]. On the one hand, there is evidence supporting that B vitamins, including vitamin B6, may help reduce the risk of stroke. However, their effectiveness may be influenced by various factors such as the patient’s underlying health condition, dosage, and combination with other supplements. On the other hand, our study’s findings should be interpreted with caution due to the limited sample size and the restricted number of SNPs associated with vitamin B6 that were analyzed. Therefore, the observed associations for genetically predicted vitamin B6 require further validation through larger and more comprehensive studies.

Nevertheless, our study has several limitations. First, even though the largest known GWAS dataset for nutrients was used to assess associations, the sample size for some nutrients was limited, possibly leading to weak instrument bias and biased estimates. However, our IVs met the criterion with F-statistics above 10, indicating that weak instrument bias was less likely to affect our results. Furthermore, a pleiotropy test could not be performed due to the small number of SNPs, with several exposures having fewer than three genome-wide significant SNPs. Second, partial results showed MR-Egger estimation with large confidence intervals. This led to less precise and unstable estimates, particularly when sample sizes were inadequate or the number of IVs was limited. Thus, we set the estimation directions of the IVW, weighted median, and MR-Egger methods to be consistent to guarantee the robustness of the results. Third, since our study focused on European populations, the results may not be fully applicable to other populations, indicating a need for cautious extrapolation and further research across diverse demographic groups. Furthermore, our findings were based on summary-level data rather than individual-level data, which limited our ability to adjust for potential confounders that could affect the exposure–outcome relationship. Future studies with larger sample sizes, diverse populations, and more sophisticated MR methods are needed to validate our findings and address these limitations comprehensively.

## 5. Conclusions

Our MR study elucidated the importance of magnesium, vitamin C, and vitamin B9 in various types of stroke prevention, providing evidence for the causal risk factors and pathological pathways of stroke. These results encourage an integrated and precise approach to dietary behavior, targeting these specific nutrients to effectively reduce the burden of stroke.

## Figures and Tables

**Table 1:** The GWAS data used in the study.

Trait	No. of SNP	Ancestry	Sample Size	Data Source
Macronutrients				
Protein	7	European	268,922	Meddens, S.F.W. et al. (2021) [11]
Carbohydrate	13	European	268,923	Meddens, S.F.W. et al. (2021) [11]
Sugar	10	European	230,648	Meddens, S.F.W. et al. (2021) [11]
Fat	6	European	268,924	Meddens, S.F.W. et al. (2021) [11]
Micronutrients				
Vitamin A	2	European	5006	Mondul, A.M. et al. (2011) [12]
Vitamin B6	2	European	4763	Hazra, A. et al. (2009) [13]
Vitamin B9	2	European	37,341	Grarup, N. et al. (2013) [14]
Vitamin B12	11	European	45,576	Grarup, N. et al. (2013) [14]
Vitamin C	11	European	52,018	Zheng, J.S. et al. (2021) [15]
Vitamin D	138	European	443,734	Manousaki, D. et al. (2020) [16]
Vitamin E	3	European	7781	Major, J.M. et al. (2011) [17]
Calcium	7	European	39,400	O’Seaghdha, C.M. et al. (2013) [18]
Copper	2	European	2603	Evans, D.M. et al. (2013) [19]
Zinc	3	European	2603	Evans, D.M. et al. (2013) [19]
Magnesium	6	European	23,829	Meyer, T.E. et al. (2010) [20]
Iron	14	European	163,511	Bell, S. et al. (2021) [21]
Selenium	12	European	9639	Cornelis, M.C. et al. (2015) [22]
Phosphorus	5	European	21,708	Kestenbaum, B. et al. (2010) [23]
Stroke				
Any stroke	-	European	446,696	Malik, R. et al. (2018) [1]
Ischemic stroke	-	European	440,328	Malik, R. et al. (2018) [1]
Large-artery stroke	-	European	150,765	Malik, R. et al. (2018) [1]
Small-vessel stroke	-	European	198,048	Malik, R. et al. (2018) [1]
Cardioembolic stroke	-	European	211,763	Malik, R. et al. (2018) [1]

## Data Availability

The original contributions presented in the study are included in the article/Supplementary Materials; further inquiries can be directed to the corresponding author.

## Supplementary Materials

The following supporting information can be downloaded at: https://www.mdpi.com/article/10.3390/nu16172818/s1, Table S1: Detailed information on instrumental variables of nutrients on stroke; Table S2: Causal estimations of nutrients on stroke in three MR methods.
